# Controlling of growth performance, lipid deposits and fatty acid composition of chicken meat through a probiotic, *Lactobacillus johnsonii* during subclinical *Clostridium perfringens* infection

**DOI:** 10.1186/s12944-017-0408-7

**Published:** 2017-02-10

**Authors:** Hesong Wang, Xueqin Ni, Lei Liu, Dong Zeng, Jing Lai, Xiaodan Qing, Guangyao Li, Kangcheng Pan, Bo Jing

**Affiliations:** 1 Animal Microecology Institute, College of Veterinary, Sichuan Agricultural University, Chengdu, 611130 China; 2 Key Laboratory of Animal Disease and Human Health of Sichuan Province, Chengdu, Sichuan China; 3 Ya’an Agricultural Science & Technology Development Co., Ltd, Ya’an, China

**Keywords:** *Clostridium perfringens*, *Lactobacillus johnsonii*, Growth performance, Meat quality, Lipid contents, Fatty acid composition

## Abstract

**Background:**

Meat is considered as a major source of polyunsaturated fatty acid (PUFA) which is essential for humans, therefore its lipid level and fatty acid composition have drawn great attention. As no clinical sign can be found in chicks subclinically infected by *Clostridium perfringens* (CP), the meat may be purchased and eaten. The objective of the present study was to determine whether *Lactobacillus johnsonii* (LJ) can control the CP-caused impact on growth, lipid levels, fatty acid composition and other flavor or nutritional quality in the meat.

**Methods:**

480 one-day-old chicks were divided into four groups and fed with basal diet (control and CP group). Supplemented with 1 × 10^5^ (L-LJ) and 1 × 10^6^ (H-LJ) colony-forming unit (cfu), CP diet was fed for 42 days. From day 19 to 22, birds of CP and LJ groups were administered with CP twice per day and the control was administered with liver broth.

**Results:**

LJ-treated chickens were free from negative influences on growth performance and significant decrease of abdominal fat deposit., LJ inhibited CP-caused shearing force and drip loss increase and pH 40 min and 24 h decrease after sacrifice. In addition, LJ exhibited a positive effect on muscle lipid peroxidation by significantly increasing SOD, CAT and GSH-Px activity and decreasing MDA level. Besides, LJ attenuated the decrease of intramuscular fat, total cholesterol and triglyceride contents caused by CP infection. However, levels of total protein and most of amino acids were not changed. CP infection decreased C18:3n-3 (α-LA), C20:4n-6, C20:5n-3(EPA), C22:4n-6, C22:5n-3, C22:6n-3(DHA), total PUFA, n-3 PUFA and PUFA:SFA ratio and increased C14:0, total SFA and n-6:n-3 ratio. LJ was found to protect the muscle from these changes. Meanwhile, the 28-day gut permeability level was higher in CP group.

**Conclusions:**

These findings suggest that CP may affect the growth performance of chicks and negatively influence lipid content and fatty acid composition in chicken meat. Meanwhile, LJ treatment may be effective in controlling these changes by reducing the increased gut permeability caused by CP subclinical infection.

## Background

Meat is a major source for humans to intake PUFAs, especially n-3PUFAs, including C18:3n-3 (α-LA), C20:5n-3 (EPA) and C22:6n-3 (DHA), which are reported to be beneficial for brain, retina, cardiovascular disease etc. [1–5]. Due to the improved living standard, there has been a considerable increase in demand for better safety and quality meat in recent years [6, 7]., As a gram-positive anaerobic spore-forming, rod-shaped bacterium, *Clostridium. perfringens* (CP) has posed an important health risk to chickens all over the world [8]. Relevant researches usually focus on necrotic enteritis, an enterotoxemic disease caused by CP, leading to necrotic lesions development in the gut wall and a number of clinical signs, such as depression, dehydration, diarrhea, ruffled feathers and poultry mortality [9, 10]. However, the subclinical form of CP infection also damages the intestinal tract and presents as poor performance and reduced feed conversion without mortality. Since no clinical sign can be found in CP-caused subclinical infection, the prevention is more difficult and most economic losses are associated with the subclinical form [11], or even worse, meat of infected birds may be purchased and eaten by customers. CP-induced necrotic enteritis has been found to significantly lower high-density lipoprotein cholesterol (HDL-C) in the serum and inhibit mRNA expression of peroxisome proliferator-activated receptor α (PPARα) and carnitine palmitoyl transferase-1 (CPT-1) in the liver of broilers, which may cause an impact on the body lipid metabolism [12]. Besides, CP may influence lipid metabolism by damaging intestinal absorption. Unfortunately, there is no relevant research regarding lipid changes in chicken under the CP-caused subclinical infection. Therefore, the present study aimed to evaluate the CP-caused impact on growth performance, lipid content, fatty acid composition and other flavor or nutritional substances in the meat. As the application of probiotics is becoming a common method to prevent CP in post-antibiotic era [13, 14], the present study also aimed to determine whether the dietary supplemented probiotic, *Lactobacillus johnsonii* (LJ), can control these potential changes. *Lactobacillus johnsonii* BS15 (CCTCC M2013663) is isolated from homemade yogurt collected from the Hongyuan Prairie, Aba Autonomous Prefecture, China, which can prevent non-alcoholic fatty liver disease by attenuating inflammation and mitochondrial injury and improving gut environment in obese mice [15].

## Results

### Growth performance and carcass traits

As shown in Table 1, significant increase in FCR and decrease (*P* < 0.05) in final weight, DWG, breast percentage and thigh percentage were observed in CP group when compared with control. FCR was significantly lower and final weight and DWG were significantly higher (*P* < 0.05) in H-LJ group when compared with CP group. In CP group, abdominal fat level, and values of breast percentage and thigh percentage were significantly lower than those in other three experimental groups (*P* < 0.05). However, there was no difference in FI and survival rate among the four groups (*P* > 0.05).

**Table 1:** Effect of *L. johnsonii* on growth performance and carcass traits of CP-induced chickens^1^

Item^2^	Control	CP	L-LJ	H-LJ	Pooled SE	*P*-value
Initial weight, g	41.5	41.7	42.0	41.9	0.38	0.951
Final weight, g	2512.7^a^	2214.1^c^	2307.8^bc^	2399.8^b^	28.81	<0.001
DWG, g.d^−1^	58.8^a^	51.7^c^	53.9^bc^	56.1^b^	0.68	<0.001
FI, g	4410.7	4297.5	4363.2	4382.9	19.58	0.212
FCR	1.78^c^	1.97^a^	1.93^a^	1.86^b^	0.02	0.002
Survival rate, %	96.52	94.12	95.20	95.08	0.32	0.053
Abdominal fat, %^3^	1.21^a^	1.12^b^	1.18^a^	1.17^a^	0.01	0.005
Breast, %^3^	7.45^a^	6.72^c^	7.01^b^	7.07^b^	0.05	<0.001
Thigh, %^3^	9.26^a^	8.69^c^	8.99^b^	9.04^b^	0.04	<0.001

^a,b^ Means in the same row with different superscripts are significantly different. (*P* < 0.05)

^1^control = basal diet + PBS; CP = basal diet + CP administration; L-LJ = basal diet with 1.0 × 10^5^ cfu BS15/g diet + CP administration; H-LJ = basal diet with 1.0 × 10^6^ cfu BS15/g diet + CP administration. Pen (n = 6 per treatment group) was used as the experimental unit for initial weight, final weight, DWG, FI, FCR and survival rate. An individual bird (n = 12 per treatment group) was used as the experimental unit for abdominal fat, breast and thigh percentages

^2^ *DWG* daily weight gain, *FI* feed intake, *FCR* feed conversion ratio (FI/body weight gain)

^3^% = percentage of 42-day body weight

### Meat color, pH, drip loss and shearing force

As shown in Table 2, no differences in meat color values were detected (*P* > 0.05), including a^*^, b^*^ and L^*^. When compared with the control, significantly higher drip loss and shearing force and lower pH40min and pH24h (*P* < 0.05) were observed in CP group. Except the breast pH24h in L-LJ group, pH40min and pH24h of breast and thigh muscles were significantly higher (*P* < 0.05) in two LJ groups than those in CP group. In H-LJ group, lower drip loss and shearing force values of breast and thigh muscles were observed when compared with CP group (*P* < 0.05).

**Table 2:** Effect of *L. johnsonii* on meat quality of CP-induced chickens^1^

Item^2^	Control	CP	L-LJ	H-LJ	Pooled SE	*P*-value
Breast						
a^*^	1.41	1.44	1.41	1.39	0.02	0.872
b^*^	4.74	4.64	4.69	4.68	0.08	0.745
L^*^	46.96	48.91	46.69	45.42	0.56	0.172
pH 40 min	5.89^a^	5.56^c^	5.72^b^	5.72^b^	0.03	<0.001
pH 24 h	5.51^a^	5.24^c^	5.33^bc^	5.46^a^	0.02	<0.001
Drip loss, %	1.77^c^	1.92^a^	1.88 ^ab^	1.81^bc^	0.02	0.001
Shearing force, kg	2.69^c^	2.88^a^	2.81^ab^	2.70^bc^	0.02	0.001
Thigh						
a^*^	1.53	1.51	1.49	1.54	0.01	0.806
b^*^	4.83	4.77	4.79	4.68	0.03	0.386
L^*^	45.92	47.71	46.28	46.33	0.341	0.265
pH 40 min	5.63^a^	5.19^c^	5.41^b^	5.55^a^	0.03	<0.001
pH 24 h	4.89^a^	4.44^d^	4.61^c^	4.72^b^	0.03	<0.001
Drip loss, %	1.70^c^	1.88^a^	1.83 ^ab^	1.78^b^	0.01	<0.001
Shearing force, kg	2.77^c^	2.95^a^	2.88^ab^	2.81^bc^	0.02	<0.001

^a,b^Means in the same row with different superscripts are significantly different. (*P* < 0.05)

^1^control = basal diet + PBS; CP = basal diet + CP administration; L-LJ = basal diet with 1.0 × 10^5^ cfu BS15/g diet + CP administration; H-LJ = basal diet with 1.0 × 10^6^ cfu BS15/g diet + CP administration

^1^n = 12 per treatment group

^2^a^*^ = redness; b^*^ = yellowness; L^*^ = lightness

### Muscle antioxidant ability

As shown in Table 3, no significant changes of SOD and GSH-Px were observed (*P* > 0.05) among various treatment groups. However, the CAT activity was lower and MDA content was higher in CP group when compared with the control (*P* < 0.05). Furthermore, the CAT activity was significantly higher (*P* < 0.05) and MDA content of breast and thigh muscle was significantly lower (*P* < 0.05) in both L-LJ and H-LJ groups than those in CP group.

**Table 3:** Effect of *L. johnsonii* on muscle antioxidant ability of CP-induced chickens^1^

Item	Control	CP	L-LJ	H-LJ	Pooled SE	*P*-value
Breast						
SOD, U/mg protein	51.33	49.28	50.08	50.01	0.31	0.137
CAT, U/mg protein	6.31^a^	5.88^c^	6.01^b^	6.20^a^	0.03	<0.001
GSH-Px, U/mg protein	59.07	56.08	57.22	58.01	0.63	0.395
MDA, nmol/mg protein	1.61^c^	1.92^a^	1.75^b^	1.69^bc^	0.02	<0.001
Thigh						
SOD, U/mg protein	54.76	53.22	54.11	53.98	0.38	0.559
CAT, U/mg protein	6.66^a^	6.15^c^	6.33^b^	6.31^b^	0.03	<0.001
GSH-Px, U/mg protein	66.77	64.98	65.08	65.11	0.28	0.066
MDA, nmol/mg protein	1.44^c^	1.79^a^	1.61^b^	1.62^b^	0.02	<0.001

^a,b^Means in the same row with different superscripts are significantly different. (*P* < 0.05)

^1^control = basal diet + PBS; CP = basal diet + CP administration; L-LJ = basal diet with 1.0 × 10^5^ cfu BS15/g diet + CP administration; H-LJ = basal diet with 1.0 × 10^6^ cfu BS15/g diet + CP administration

^1^n = 12 per treatment group

### Total protein, intramuscular fat, inosine monophosphate, total cholesterol and triglyceride levels

Results in Table 4 showed that IMF, TC and TG contents were significantly decreased (*P* < 0.05) in CP group when compared with the control. However, total protein and IMP did not change (*P* > 0.05). In addition, higher IMF and TC contents were observed (*P* < 0.05) in L-LJ and H-LJ groups in comparison with CP group.

**Table 4:** Effect *L. johnsonii* on muscle total protein, IMF, IMP, TC and TG contents of CP-induced chickens^1^

Item	Control	CP	L-LJ	H-LJ	Pooled SE	*P*-value
Breast						
Total protein, %	20.51	19.95	21.11	20.08	0.23	0.292
IMF, %	2.09^a^	1.72^c^	1.88^b^	1.91^b^	0.02	<0.001
IMP, mg/g	2.53	2.51	2.53	2.57	0.01	0.444
TC, mg/g	0.88^a^	0.56^c^	0.69^b^	0.73^b^	0.02	<0.001
TG, mg/g	0.45^a^	0.31^c^	0.33^c^	0.38^b^	0.01	<0.001
Thigh						
Total protein, %	19.71	19.51	19.55	20.01	0.13	0.517
IMF, %	1.88^a^	1.61^c^	1.70^b^	1.70^b^	0.02	<0.001
IMP, mg/g	2.62	2.64	2.64	2.55	0.02	0.245
TC, mg/g	0. 66^a^	0.41^c^	0.55^b^	0.55^b^	0.02	<0.001
TG, mg/g	0.37^a^	0.29^b^	0.31^b^	0.33^ab^	0.01	0.006

^a,b^Means in the same row with different superscripts are significantly different. (*P* < 0.05)

^1^control = basal diet + PBS; CP = basal diet + CP administration; L-LJ = basal diet with 1.0 × 10^5^ cfu BS15/g diet + CP administration; H-LJ = basal diet with 1.0 × 10^6^ cfu BS15/g diet + CP administration

^1^ *IMF* intramuscular fat, *IMP* inosine monophosphate, *TC* total cholesterol, *TG* triglyceride

^1^n = 12 per treatment group

### Amino acid composition in breast muscle

As shown in Table 5, AAs content did not change (*P* > 0.05), except that Cys content in CP group significantly decreased when compared with the other three groups (*P* < 0.05) and EAA content in CP group was significantly lower than that in the control (*P* < 0.05). In LJ groups, EAA content was higher than that in CO group, which however was limited (*P* > 0.05).

**Table 5:** Effect of *L. johnsonii* on amino acid composition (g/kg) in breast muscle of CP-induced chickens^1^

Item^2^	Control	CP	L-LJ	H-LJ	Pooled SE	*P*-value
Aspartic acid (Asp)	22.20	23.10	22.84	23.27	0.26	0.503
Threonine (Thr)	8.47	8.32	8.31	8.40	0.10	0.935
Serine (Ser)	7.81	8.01	7.88	7.80	0.11	0.915
Glutamic acid (Glu)	39.91	39.07	39.67	40.01	0.59	0.947
Glycine (Gly)	5.66	5.41	5.56	5.61	0.09	0.774
Alanine (Ala)	9.51	9.17	9.22	9.33	0.15	0.862
Cystine (Cys)	1.72^a^	1.44^b^	1.68^a^	1.66^a^	0.03	0.001
Valine (Val)	12.14	11.94	12.08	12.04	0.20	0.988
Methionine (Met)	5.54	5.22	5.30	5.39	0.07	0.359
Isoleucine (Ile)	13.17	11.92	12.82	12.74	0.19	0.116
Leucine (Leu)	23.52	21.89	22.07	22.84	0.37	0.387
Tyrosine (Tyr)	4.01	3.89	3.91	4.02	0.05	0.723
Phenylalanine (Phe)	9.77	9.74	9.78	9.71	0.13	0.998
Lysine (Lys)	19.99	20.04	19.85	19.88	0.27	0.994
Histidine (His)	10.71	9.94	9.91	10.08	0.16	0.272
Arginine (Arg)	16.71	16.21	15.93	16.08	0.16	0.347
Proline (Pro)	7.81	7.62	7.71	7.77	0.11	0.932
EAA	127.83^a^	122.83^b^	123.76^b^	124.91^ab^	0.65	0.035
FRAA	103.53	102.01	102.61	103.73	0.62	0.749
Total	218.66	212.91	214.52	216.61	0.99	0.189

^a,b^Means in the same row with different superscripts are significantly different. (*P* < 0.05)

^1^control = basal diet + PBS; CP = basal diet + CP administration; L-LJ = basal diet with 1.0 × 10^5^ cfu BS15/g diet + CP administration; H-LJ = basal diet with 1.0 × 10^6^ cfu BS15/g diet + CP administration

^1^n = 12 per treatment group

^2^ *EAA* essential amino acid (including threonine, valine, methionine, isoleucine, leucine, phenylalanine, lysine, histidine, arginine, and proline), *FRAA* flavor-related amino acids (including cystine, glycine, aspartic acid, arginine, proline, alanine, and glutamic acid)

### Fatty acid composition in breast muscle

As shown in Table 6, C14:0 and total SFA contents and n-6:n-3 ratio in CP group were significantly higher (*P* < 0.05) than those in the control and some of them were also higher than those in the LJ groups, especially in the H-LJ group (*P* < 0.05). In contrast, α-LA, C20:4n-6, EPA, C22:4n-6, C22:5n-3, C22:6n-3 (DHA), total PUFA and n-3 PUFA contents and PUFA:SFA ratio were significantly lower (*P* < 0.05) than those in the control and some of them were also lower than those in the LJ groups, including C20:4n-6, EPA, DHA, total PUFA and n-3 PUFA contents and PUFA:SFA ratio.

**Table 6:** Effect of *L. johnsonii* on fatty acid composition (mg/100 g of dried meat) in breast muscle of CP-induced chickens^1^

Item^2^	Control	CP	L-LJ	H-LJ	Pooled SE	*P*-value
C14:0	24.4^c^	28.14^a^	26.24^b^	25.55^bc^	0.33	<0.001
C16:0	724.7	771.2	759.2	758.1	7.38	0.136
C16:1n-7	131.2	140.6	133.5	135.8	2.22	0.495
C18:0	311.9	341.2	340.7	329.5	5.22	0.158
C18:2n-6	909.4	862.4	888.2	884.3	11.91	0.594
C18:3n-3 (α-LA)	35.71^a^	31.22^c^	32.81^b^	33.92^ab^	0.45	0.002
C20:2n-6	14.91	14.27	14.29	15.01	0.16	0.220
C20:4n-6	111.8^a^	88.24^c^	91.21^bc^	99.34^b^	2.07	<0.001
C20:5n-3 (EPA)	9.99^a^	6.28^c^	7.91^b^	8.22^b^	0.22	<0.001
C22:4n-6	19.71^a^	15.27^b^	15.98^b^	16.29^b^	0.38	<0.001
C22:5n-3	33.91^a^	27.57^b^	29.33^b^	29.77^b^	0.47	<0.001
C22:6n-3 (DHA)	7.07^a^	6.02^b^	6.55^ab^	6.93^a^	0.12	0.004
Total SFA	1297^b^	1455^a^	1391^ab^	1329^b^	18.16	0.008
Total MUFA	1301	1202	1255	1257	17.38	0.250
Total PUFA	1,008^a^	901.5^c^	951.2^bc^	977.3^b^	12.94	0.022
n-6 PUFA	917.4	878.6	899.6	901.4	9.56	0.571
n-3 PUFA	81.21^a^	67.07^c^	74.31^b^	75.29^b^	0.99	<0.001
PUFA:SFA ratio	0.78^a^	0.62^c^	0.68^bc^	0.74^ab^	0.02	0.002
n-6:n-3 ratio	11.30^b^	13.10^a^	12.11^b^	11.97^b^	0.19	0.004

^a,b^Means in the same row with different superscripts are significantly different. (*P* < 0.05)

^1^control = basal diet + PBS; CP = basal diet + CP administration; L-LJ = basal diet with 1.0 × 10^5^ cfu BS15/g diet + CP administration; H-LJ = basal diet with 1.0 × 10^6^ cfu BS15/g diet + CP administration

^1^n = 12 per treatment group

^2^ *SFA* saturated fatty acid, *MUFA* monounsaturated fatty acid, *PUFA* polyunsaturated fatty acid

### Gut permeability

**Fig. 1:**
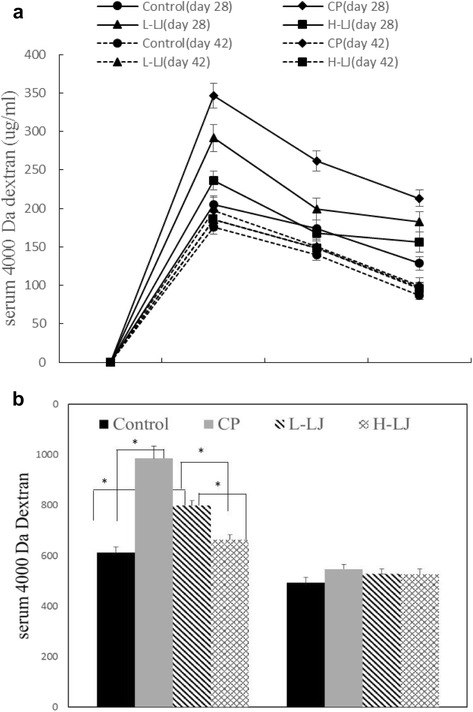
The effect of *L. johnsonii* on the gut permeability of CP-induced chickens. **a** Intestinal permeability assay—serum 4000 Da dextran (μg/ml) oral challenge measured in chickens from control, CP, L-LJ, and H-LJ groups at 28 and 42 days. **b** The area under curve corresponding to a in the same groups.*P < 0.05

Figure 1b shows that under the curve of serum 4000 Da dextran, the CP group revealed a higher area at day 28 when compared with the control group (*P* < 0.05), indicating enhanced gut permeability and LJ groups showed a lower area (*P* < 0.05) under the curve of serum 4000 Da dextran when compared with the CP group. However, area under the curve of serum 4000 Da dextran of CP group is higher than that in the other three groups, but not significant at day 42 (*P* > 0.05).

## Discussion

Along with the ban of antimicrobial growth promoters, CP infection is of significant economic consequence to the Poultry Industry, especially the subclinical form [11]. Chicken meat is considered to be one of the main sources of PUFA, especially n-3 PUFA and its FA composition is proved to be modulated by altering lipid intake and absorption levels [16, 17]. As shown in previous studies, CP not only damages small intestine [10, 18], it is also associated with hepatitis or cholangiohepatitis [19]. Therefore, CP may impact the lipid metabolism of broilers and thus cause a side effect on FA composition and other flavour or nutritional substances of chicken meat. The present study revealed that subclinical CP infection strongly reduced the growth performance of broilers and changed flavour and nutritional substances in the meat, especially values relating to lipid metabolism. As live cultures of beneficial bacteria that equilibrate intestinal microflora to benefit the host, probiotics are considered to be the most desirable alternative to antibiotic growth promoters that are prohibited in livestock [20, 21]. In recent years, great effort in animal feed research has been directed toward the application of probiotics to obtain high-quality chicken meat [22, 23] and control avian diseases, including CP infection [24, 25]. As shown in this study, positive effects were observed when different concentrations of LJ were used to control CP infection.

Although DWG and FCR changed significantly, no difference in survival rate was observed among four groups. In this study, the characteristic of CP group was in accordance with the description of subclinical CP infection [26]. Moreover, the results showed that FCR significantly changed, however no differences in FI were observed among the groups, suggesting that FCR improvement might be due to nutrient digestibility of broilers [27]. In the present study, abdominal fat and breast and thigh percentages in LJ groups were markedly increased when compared with CP group, suggesting that LJ may be beneficial for broilers malnutrition caused by CP, although abdominal fat of broilers is considered to be an added waste for treating effluent from processing.

The results presented significantly lower pH values (both 40 min and 24 h after sacrifice), which indicated a higher postmortem acidification rate [28]. pH values of breast and thigh meat in LJ groups were markedly higher than those in CP group, suggesting a positive influence of LJ on prolonging meat shelf life. Interestingly, no obvious changes in the meat color were detected in this study, which was inconsistent with similar reports concerning chicken meat [29, 30]. Consequently, unchanged meat color may make infected meat more difficult to be distinguished. No significant difference in SOD and GSH-Px activities was observed among the four experimental groups, whereas MDA levels were lower in the LJ groups than that in the CP groups. MDA is a lipid peroxidation index, which reflects tissue oxidative damage, one of the major causes of quality deterioration in meat [31, 32]. Therefore, the lowering trend of MDA affected by LJ showed a positive influence on prolonging meat shelf life against the damage of CP infection.

In this study, the majority of values relating to protein and amino acid composition remained unchanged, as well as the IMP value which is one of the main umami compounds in poultry meat. Nevertheless, when compared with CP group, IMF, TG and TC of breast and thigh muscles were significantly increased in LJ groups, which however remained lower than those in the control group. Similar results were reported for the effect of a probiotic, *Rhodobacter capsulatus*, on cholesterol concentration [22]. As reported by Skrivan et al. [33], cholesterol concentration modulation by dietary means was more difficult than the modulation of fatty acid composition of poultry meat. It was suggested that probiotics may be effective in regulating TC and TG levels by improving gut microbiota and thereby modulating digestive mechanisms. Results revealed that FA composition of breast muscle was changed significantly by CP and LJ was found to protect muscle from these changes. As one of the most important nutrition substances, n-3 PUFA in broiler meat ameliorates a number of human diseases, especially cardiovascular disease [4]. Meanwhile, positive effects of n-3 PUFA on bronchial asthma, neuropsychiatric disorders and cognitive brain function in childhood were demonstrated and n-3 PUFA could also prevent future cardiovascular events [34]. However, the population showed a large deficit for n-3 PUFA, while the overall n-6 PUFA intake exceeded the upper level [35]. Previous reports of n-3 PUFA have mainly focused on the benefits of DHA and EPA, which have important effects on retina and brain [3, 36, 37]. Furthermore, DHA and EPA are reported to reduce oxidative stress in type 2 diabetic patients [38]. In our study, n-3 PUFA values were significantly decreased, including α-LA, EPA and DHA and n-6:n-3 ratio was higher in CP group when compared with the control, suggesting nutrition destruction in the meat. In addition, PUFA changes are consistent with present findings of CAT activity and MDA level, since it is reported that PUFA is easy to be oxidized [39]. Besides, the results showed that side effects of CP infection on the FA composition were effectively controlled by LJ, which was in agreement with previous studies, in which probiotics were used to improve FA composition of meat [29, 40]. However, the mechanism by which probiotics improve meat quality is still unknown. Gut permeability was also increased in CP group. Under LJ treatment, serum 4000 Da dextran levels at day 28 were markedly decreased, while no significant difference was observed among the four groups at day 42 . The results indicated that CP could impact gut permeability and intestine damage gradually recovered according to the extension of time. As reported by Krogdahl [41], the ability to digest lipid is not fully developed in the very young bird and therefore influences of lipid deposit and FA composition caused by CP may be due to the increased gut permeability. As a probiotic, LJ may also protect broilers from subclinical CP infection by controlling abnormally increased gut permeability.

## Conclusion

In conclusion, CP may impact the growth performance of chicks and damage lipid content and fatty acid composition in chicken meat. Meanwhile, LJ treatment may be effective in controlling these changes by reducing the increased gut permeability caused by subclinical CP infection.

## Methods

### Material

A total of 480 one-day-old male chicks (Cobb 500) with similar body weight were attained from Chia Tai broiler hatchery, Chengdu, China. Chicks were weighed and allotted into 4 treatment groups consisting of 6 replicates with 20 birds per replicate at the Key Laboratory of Animal Disease and Human Health of Sichuan Province, Sichuan Agricultural University. The amounts of BS15 cells were evaluated by heterotrophic plate counts after maintaining the cultures in MRS broth at 37 °C for 36 h under anaerobic environment. Then the bacterial cells were collected, washed with saline, and then suspended in phosphate buffered saline (PBS) (pH 7.0) for experimental use.

The temperature of the room was maintained at 33 °C for the first 3 days, after which the temperature was gradually reduced by 3 °C a week until reaching 24 °C. The temperature of the room was then maintained at 24 °C for the remainder of the experiment. Artificial light was provided 24 h/day by the use of fluorescent lights. The starter and finisher diet formula is shown in Table 7. All diets were formulated to meet or exceed the NRC (1994) requirements for broilers. The 4 groups of birds were fed as follows: control (basal diet), CP Group (basal diet), L-LJ Group (basal diet + 1× 10^5^ cfu BS15/g as fed) and H-LJ Group (basal diet + 1× 10^6^ cfu BS15/g as fed).

**Table 7:** Composition of the basal diets for chickens

Ingredient^a^	Starter diet (%) 1 to 21d	Finisher diet (%) 22 to 42 d
Ground yellow corn	56.0	59.5
Soybean meal	37.0	32.85
Soybean oil	3.66	4.7
Ground limestone	0.57	0.5
Dicalcium phosphate	1.8	1.6
Salt	0.3	0.3
Choline chloride	0.1	0.1
DL-Methionine	0.24	0.12
Micronutrients^b^	0.33	0.33
Calculated nutrients level (%)		
Metabolic energy (MJ kg-1)	12.39	12.79
Crude protein	21.17	19.72
Lys	1.19	1.08
Met	0.50	0.40
Met + Cys	0.86	0.74
Ca	0.85	0.77
Nonphytate P	0.44	0.40

^a^Ingredient and nutrient composition are reported on as-fed basis

^b^Micronutrients are provided per kilogram of diet: vitamin A (all-trans retinol acetate), 12 500 IU; cholecalciferol, 2 500 IU; vitamin E (all-rac-a-tocopherol acetate), 18.75 IU; vitamin K (menadione Na bisulfate), 5.0 mg; thiamin (thiamin mononitrate), 2.5 mg; riboflavin, 7.5 mg; vitamin B6, 5.0 mg; vitamin B12, 0.0025 mg; pantothenate, 15 mg; niacin, 50 mg; folic acid, 1.25 mg; biotin, 0.12 mg; Cu (CuSO_4_ · 5H_2_O), 10 mg; Mn (MnSO_4_ · H_2_O), 100 mg; Zn (ZnSO_4_ · 7H_2_O), 100 mg; Fe (FeSO_4_ · 7H_2_O), 100 mg; I (KI), 0.4 mg; Se (Na_2_SeO_3_), 0.2 mg

### Producers

Following the incubation, the bacteria cultures confirmed to be *C. perfringens* type A by standard biochemical tests and PCR test (alpha toxin and NetB positive), were enumerated and adjusted to a final concentration of 1.0 × 10^8^ cfu/mL, and distributed in 1 mL aliquots in glass vials. The vials were stored at −70 °C. From day 19 to 22, the birds of CP and LJ groups were orally administered with 1 mL of CP per bird. The control was administered with the same amount of liver broth instead.

The chicks were weighed and feed intake (FI) was recorded on days 1 and 42, and body weight gain (BWG) and feed conversion ratio (FCR) were then calculated from these values. Survival rate was recorded during the experimental days. On day 42, 12 birds from each source (2 birds per replicate) were randomly selected and humanely sacrificed. Thigh (*biceps femoris*), and breast (*pectoralis major*) muscles without skin were removed and weighed. Abdominal fat levels were evaluated as the percentage of carcass weight by removing and weighing all adipose tissues surrounding the gizzard, cloaca, and adjacent muscles. Percentages of breast and thigh muscle were expressed as the ratios of each of these weights to 42-day body weight. The muscles from the left sides of the breast and thigh were used to determine meat color, drip loss, pH40min and pH24h after sacrifice) and intramuscular fat (IMF) levels. Appropriate sizes of breast and thigh muscles from the right side were removed to determine shearing force, then the breast and thigh muscle from the right side were removed immediately and chilled to 0 °C. At the laboratory, the samples were frozen at −18 °C until they were analyzed.

The meat color was evaluated by measuring the L* (lightness), a* (redness), and b* (yellowness) color values using a Minolta CR-300 chromameter (Konica Minolta Sensing Inc., Osaka, Japan). The pH value of breasts was determined using a pH meter (pH 211, Hanna, Padua, Italy). The pH value was measured using a PH-STAR direct probe (Matthäus, Pöttmes, Germany) by thrusting the probe into the muscle. Drip loss was assessed from breast meat packaged in a transparent polythene bag and stored in a chilling room at 4 °C for 72 h, after which the excess moisture was wiped off and the breast samples were weighed (at 72 h). Drip loss was calculated as the difference between the breast weight measured at 0 h and 72 h, divided by breast weight at 0 h. Shearing force was measured according to the methods described by Zhuang et al. [40]. At 20 °C–25 °C, samples (strips of 19 mm width) were sheared perpendicular to the longitudinal orientation of the muscle fibers with a TA.XT Plus/50 texture analyzer (Stable Micro Systems, Surrey, UK) fitted with a 30 kg load cell and Texture Exponent 32 version 3.0.3.0 software. A TA-7 WB shear-type blade was used. The test settings included a button-type trigger, travel distance of 55 mm, test speed of 4 mm/s, and calibration return distance of 1 mm. The maximum force needed to cut the strips was expressed in kg/cm. For each breast muscle, one strip was sheared in 2 locations and the strip heights at each shear point were recorded and used for data analysis. IMF levels in the breast muscle were determined by extraction with petroleum ether (bp range 30-60 °C) in a Soxhlet apparatus, and the IMF percentage was calculated [42].

The muscles were weighed and homogenized in nine volumes of ice-cold 0.85% NaCl solution in a chilled homogenizer, and then immediately centrifuged at 3500 × g at 4 °C. The samples were used for the determination of the levels of inosine monophosphate (IMP), triglyceride (TG), total cholesterol (TC), and malondialdehyde (MDA) and activities of superoxide dismutase (SOD), catalase (CAT), and glutathione peroxidase (GSH-Px). The levels of TG and TC in the muscle samples were measured with an automatic biochemical analyzer (RA-1000, Bayer Corp., Tarrytown, NY) using colorimetric methods. IMP levels were determined by ELISA (Shanghai Hufeng Chemical Engineering Institute of China) following the manufacturer’s instruction. The MDA levels and activities of SOD, CAT and GSH-Px were determined using reagent kits (Nanjing Jiancheng Bioengineering Institute of China) following the manufacturer’s instructions.

The total protein was determined by the Kjedahl method [43]. According to the methods of Zhao et al. [44], the amino acid (AA) composition was determined on a Beckman 6300 amino acid analyzer (Beckman Instruments Corp., Brea, CA) equipped with a cation separation column (SYKAM LCAK06/Na) (4.6 × 150 mm) using ninhydrin for postcolumn derivatization and norleucine as the internal standard (Sigma, USA). Samples were hydrolyzed with 6 N HCl for 24 h at 110 °C before analysis. Methionine and cysteine were determined as methionine sulfone and cysteic acid after cold perfomic acid oxidation before hydrolysis.

Using the method of Sukhija and Palmquist [45] and Elkin [46], breast muscle samples were freeze-dried and ground for extraction and methylation of fatty acids before analysis using an HP6890 gas chromatograph equipped with a flame-ionization detector and a DB-23 capillary column (0.25 mm × 60 m × 0.25 μm; J&W Scientific, Folsom, CA). The following oven temperature program was used: 180 °C held for 10 min, increased to 220 °C at 4 °C/min held for 15 min, and increase to 250 °C at 3 °C/min. A 1-μL sample was injected with a split ratio of 1:20 at an inlet temperature of 250 °C. Heliumwas used as the carrier gas at a constant flow rate of 1.1 mL/min. Individual FAs were identified by comparison of their retention times with those in a standard mix of FAs (Supelco 37 component FAME mix), and quantification of individual fatty acids (% of total fatty acids) was performed against a C19:0 internal standard from Sigma (USA).

The measure of gut permeability is based on the methods described by Cani et al. [47]. At day 28 and 42, 6 chicks (1 bird per replicate) of each group were randomly selected and orally administered with 4000 Da dextran (Shanghai Bioleaf Biotech Co., Ltd., Shanghai, China) (500 mg/kg body weight, 125 mg/mL) after being fasted for 6 h. Blood samples were collected from the tip of the tail vein after 1, 2.5, and 4 h. The blood was centrifuged at 4 °C and 12,000 × g for 3 min, and the obtained serum was then determined using ELISA kit (Shanghai Bioleaf Biotech Co., Ltd.) according to the manufacturer’s instructions.

### Statistical analysis

Data were analyzed by one-way ANOVA using the GLM procedure of SPSS 21.0 (SPSS Inc., Chicago, IL). Differences among all treatments were evaluated using Duncan’s multiple range test. Probability values of < 0.05 were considered significant.
